# Bacterial Brain Abscess and Life-Threatening Intracranial Hypertension Requiring Emergent Decompressive Craniectomy After SARS-CoV-2 Infection in a Healthy Adolescent

**DOI:** 10.7759/cureus.36258

**Published:** 2023-03-16

**Authors:** Thitikan Kunapaisal, Shuhong Guo, Courtney Gomez, Marie A Theard, John B Lynch, Abhijit V Lele, Mary A King, Robert Buckley, Monica S Vavilala

**Affiliations:** 1 Harborview Injury Prevention and Research Center, University of Washington, Harborview Medical Center, Seattle, USA; 2 Anesthesiology and Pain Medicine, University of Washington, Harborview Medical Center, Seattle, USA; 3 Anesthesiology and Pain Medicine, Harborview Injury Prevention and Research Center, Harborview Medical Center, Seattle, USA; 4 Internal Medicine and Infectious Disease, Harborview Medical Center, Seattle, USA; 5 Neurocritical Care Service, Anesthesiology and Pain Medicine, University of Washington, Harborview Medical Center, Seattle, USA; 6 Pediatrics, University of Washington, Harborview Medical Center, Seattle, USA; 7 Neurological Surgery, University of Washington, Seattle, USA

**Keywords:** bacterial, child and adolescent, elevated intracranial pressure, infection, sars-cov-2, intracranial hypertension, brain abscess, sinusitis

## Abstract

Acute coronavirus 2 (SARS-CoV-2) infection usually results in mild symptoms, but secondary infections after SARS-CoV-2 infection can occur, particularly with comorbid conditions. We present the clinical course of a healthy adolescent with a brain abscess and life-threatening intracranial hypertension requiring emergent decompressive craniectomy after a SARS-CoV-2 infection. A 13-year-old healthy immunized male presented with invasive frontal, ethmoid, and maxillary sinusitis and symptoms of lethargy, nausea, headache, and photophobia due to a frontal brain abscess diagnosed three weeks after symptoms and 11 days of oral amoxicillin treatment. Coronavirus disease 2019 (COVID-19) reverse transcription-polymerase chain reaction (RT-PCR) was negative twice but then positive on amoxicillin day 11 (symptom day 21), when magnetic resonance imaging revealed a 2.5-cm right frontal brain abscess with a 10-mm midline shift. The patient underwent emergent craniotomy for right frontal epidural abscess washout and functional endoscopic sinus surgery with ethmoidectomy. On a postoperative day one, his neurological condition showed new right-sided pupillary dilation and decreased responsiveness. His vital signs showed bradycardia and systolic hypertension. He underwent an emergent decompressive craniectomy for signs of brain herniation. Bacterial PCR was positive for *Streptococcus intermedius*, for which he received intravenous vancomycin and metronidazole. He was discharged home on hospital day 14 without neurological sequelae and future bone flap replacement. Our case highlights the importance of timely recognition and treatment of brain abscess and brain herniation in patients with neurological symptoms after SARS-CoV-2 infection, even in otherwise healthy patients.

## Introduction

The acute coronavirus 2 (SARS-CoV-2) pandemic has caused six million deaths worldwide [1], and less than 20% of reported cases are in youth (0-17 years) [2]. During the early stages of the SARS-CoV-2 pandemic, the low numbers of confirmed cases in children and adolescents were attributed to under-testing due to asymptomatic or pre-symptomatic states [3-5]. However, over 14 million cases of SARS-CoV-2 infection have been diagnosed in children under 18 years old in the United States alone [5,6].

Children with SARS-CoV-2 have mild symptoms, a low risk of hospitalization, less need for mechanical ventilation, and a lower risk of life-threatening complications [3]. However, studies have repeatedly described moderate and severe SARS-CoV-2 infection in children and adolescents with coexisting conditions [3,7,8]. Although the coronavirus disease 2019 (COVID-19) vaccine effectively mitigates serious infection from SARS-CoV-2, there are reports of severe illness and complications, particularly in immunocompromised children and youth with comorbidities [9].

Acute neurological manifestations of SARS-CoV-2 are reported in pediatric and adult patients during and following SARS-CoV-2 infection [1]. Acute neurological symptoms range from mild sensory impairment such as anosmia or ageusia, impaired cognition, agitation, fatigue, insomnia, and headache to more severe neurological conditions like cranial nerve palsies, ataxia, neuralgia, corticospinal tract signs, encephalitis, cerebral venous thrombosis, seizures, Guillain-Barré syndrome, and stroke [10]. Moreover, immunocompromised youth and children with comorbid conditions are predisposed to severe SARS-CoV-2 infection, including synergistic fungal central nervous system infections [11,12]. Here, we report the clinical course and outcome of a 13-year-old previously healthy, COVID-19-vaccinated adolescent who developed a frontal brain abscess leading to life-threatening intracranial hypertension requiring abscess drainage and subsequent decompressive craniectomy after recent SARS-CoV-2 infection.

## Case presentation

**Figure 1:**
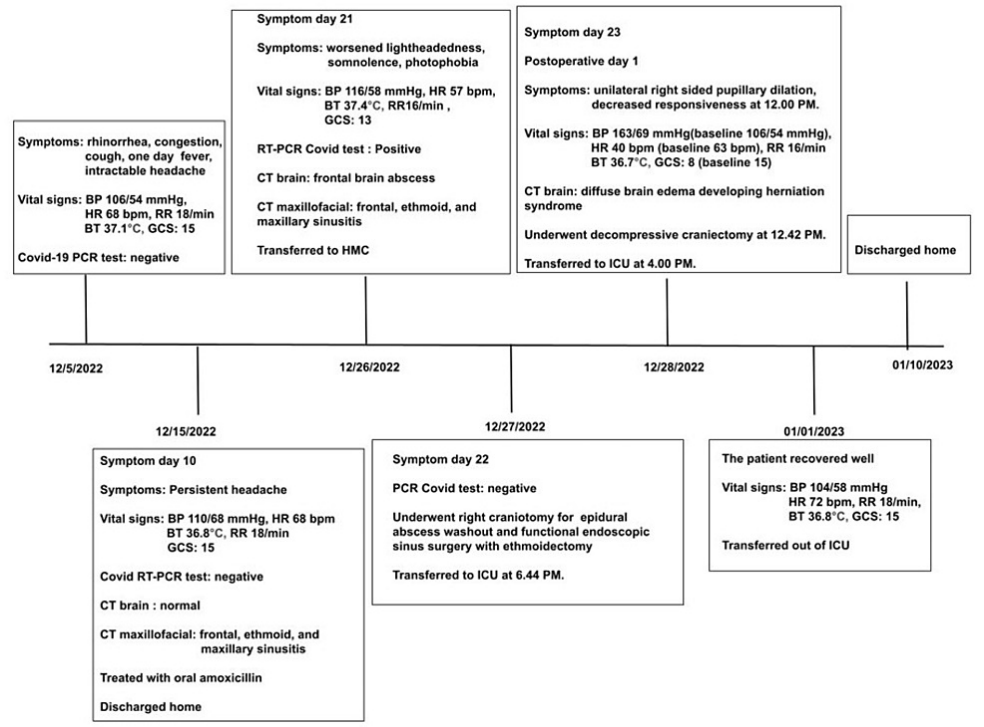
Clinical course. CT: computerized tomography; HR: heart rate; BP: blood pressure; BT: body temperature; GCS: Glasgow Coma Scale Score; bpm: beats per minute; RT-PCR: reverse transcription-polymerase chain reaction; HMC: Harborview Medical Center.

A 13-year-old healthy and fully immunized (including COVID-19) Ethiopian male patient endorsed a three-week history of rhinorrhea, congestion, cough, fever, and an intractable headache before initially seeking medical care at an outside facility. His vital signs were a blood pressure (BP) of 106/54 mmHg, a heart rate (HR) of 68 beats per minute (bpm), a respiratory rate (RR) of 18 breaths per minute, a body temperature of 37.1°C, and a Glasgow Coma Score (GCS) of 15. He was treated for migraine with supportive care, and the COVID-19 reverse transcription-polymerase chain reaction (RT-PCR) result was negative at this time (Figure 1). Family history was positive for SARS-CoV-2 infection among family members. Ten days later, he was evaluated at an outside emergency department for persistent headaches, where computed tomography (CT) of the brain was normal, and maxillofacial CT showed frontal, ethmoid, and maxillary sinusitis. His vital signs included a blood pressure of 110/68 mmHg, a heart rate of 68 beats per minute (bpm), a respiratory rate of 18 breaths per minute, a body temperature of 36.8°C, and a GCS of 15. He was discharged home with oral amoxicillin; the COVID-19 RT-PCR result was again negative. 

**Figure 2:**
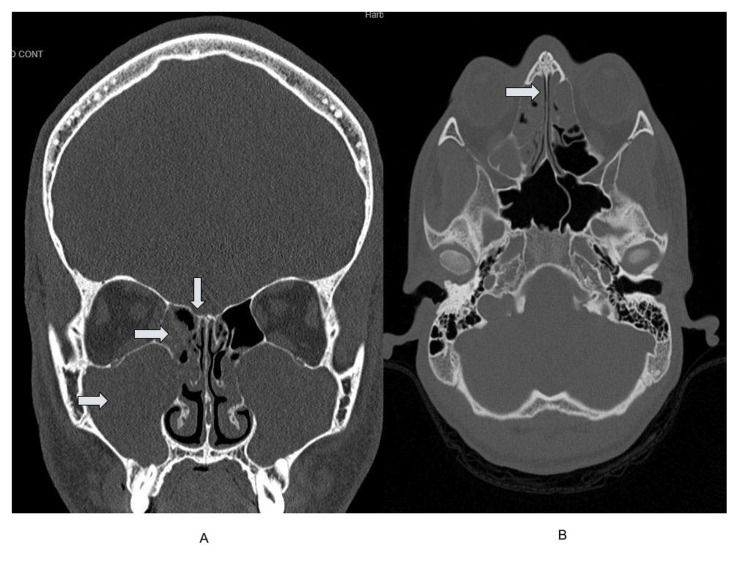
Admission (symptom day 21) computed tomography of the maxillofacial sinuses without contrast. (A) Complete opacification of both frontal and maxillary sinuses. (B) Opacification of ethmoid sinuses.

**Figure 3:**
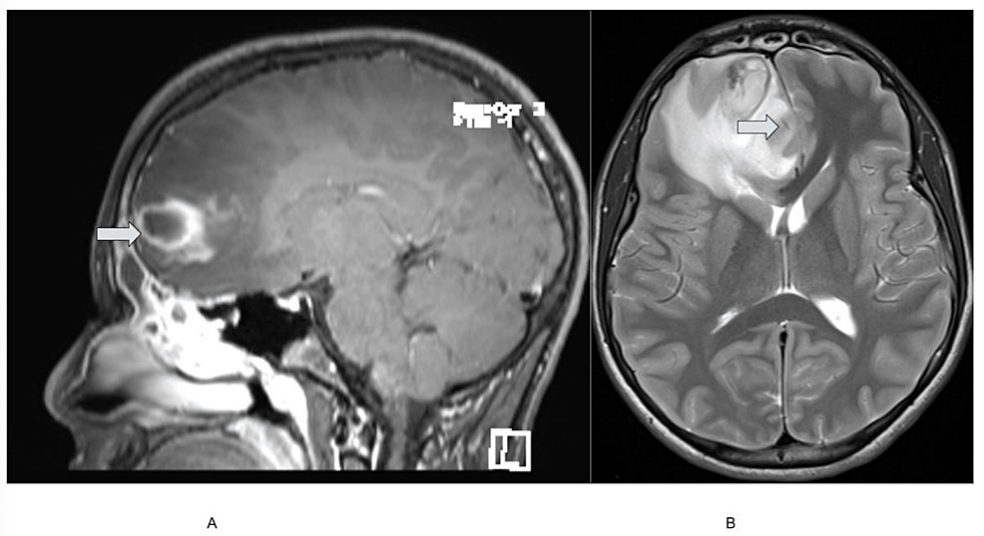
Admission (symptom day 21) magnetic resonance imaging of the brain with contrast. (A) 2.5 cm rim-enhancing fluid collection in the right frontal lobe. (B) 10 mm of anterior midline shift.

Symptoms of lightheadedness worsened over the next 11 days. On symptom day 21 (amoxicillin day 11), the patient complained of nausea and vomiting and became increasingly somnolent, for which he sought medical attention at an outside facility. His vital signs included a blood pressure of 116/58 mmHg, a heart rate of 57 bpm, a respiratory rate of 16 breaths per minute, a body temperature of 37.4°C, and a GCS of 13. CT of the brain during the third evaluation at an outside facility revealed significant vasogenic edema and a right frontal brain abscess; at this time, the COVID-19 rapid PCR result was positive. The patient was then transferred to Harborview Medical Center (HMC) for further management, where a repeat head CT confirmed frontal and maxillary sinusitis (Figure 2A) and opacification of bilateral maxillary sinuses (Figure 2B). Magnetic resonance imaging (MRI) of the brain revealed a 2.5 cm ring-enhancing fluid collection in the right frontal lobe compatible with an intraparenchymal abscess (Figure 3A) and a 10 mm anterior midline shift (Figure 3B).

Antibiotic treatment was changed to ceftriaxone 2 g intravenous (IV) every 12 h, metronidazole 600 mg IV every 8 h, and vancomycin 900 mg IV every 6 h. The patient had an emergency right craniotomy to drain an epidural abscess and functional endoscopic sinus surgery with ethmoidectomy. General anesthesia was induced with 2% lidocaine 100 mg, propofol 200 mg, fentanyl 100 mcg, and rocuronium 50 mg intravenously, and the trachea was intubated using a 7-mm, cuffed endotracheal tube. General anesthesia was maintained using 0.5 L/min air, 1.0 L/min O_2_, 2-3 vol% sevoflurane, and propofol 25-150 mcg/kg/min. Intravenous mannitol 20% (0.5 gm/kg) was administered during the procedure to attenuate brain edema. The patient’s trachea was extubated at the end of the surgery, and he was transferred to the intensive care unit. 

**Figure 4:**
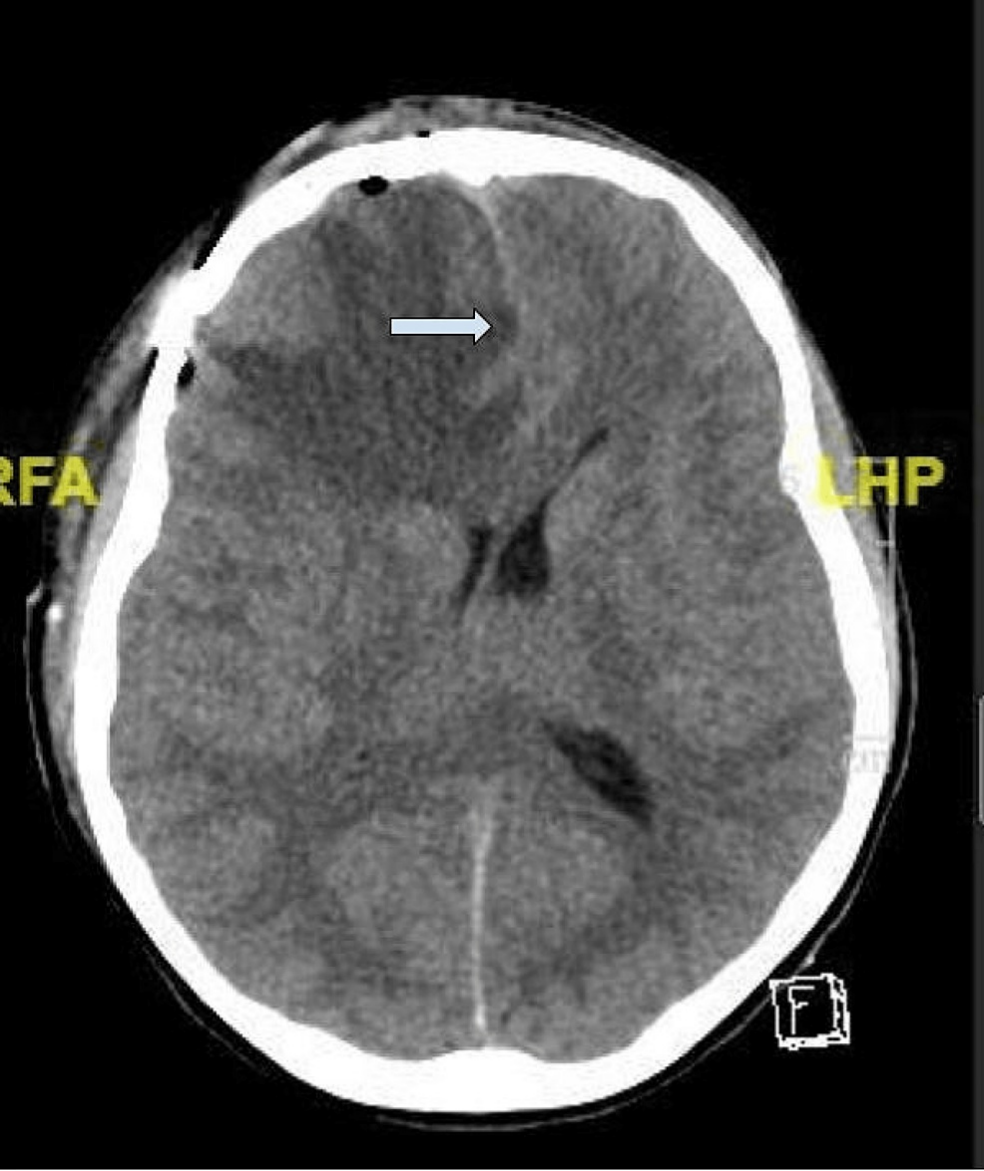
Postoperative day one computed tomography of the brain without contrast showed a mild increase in right anterior frontal lobe vasogenic edema and subfalcine herniation with a right to left midline shift before decompressive craniectomy (symptom day 23).

On a postoperative day one (symptom day 23), the patient developed new unilateral right-sided pupillary dilation and decreased responsiveness. At this time, blood pressure was 163/69 mmHg (106/54 mmHg baseline), heart rate was 40 bpm down from a baseline of 63 bpm, respiratory rate was 16 breaths per minute, and GCS was eight down from a baseline GCS of 15. Brain CT demonstrated diffuse edema with effacement of the basal cisterns and developing herniation syndrome (Figure 4), for which he underwent an emergent decompressive craniectomy. Postoperatively, the patient was admitted to a dedicated pediatric intensive care unit, remained intubated, and sedated on propofol. On post-decompressive craniectomy day one, his neurological examination improved, and he was extubated.

**Figure 5:**
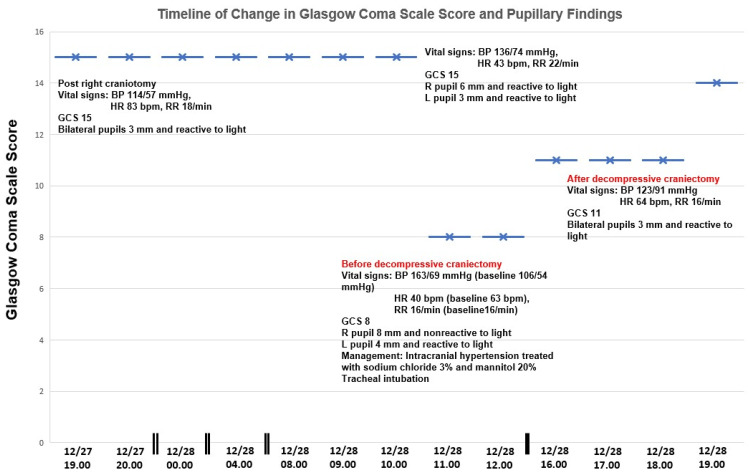
Change in Glasgow Coma Scale score and pupillary findings. GCS: Glasgow Coma Scale; BP: blood pressure; HR: heart rate; RR: respiratory rate; mm: millimeters; bpm: beats per minute.

Figure 5 highlights the neurological presentation, which includes the change in the Glasgow Coma Scale Score and pupillary examination findings.

**Figure 6:**
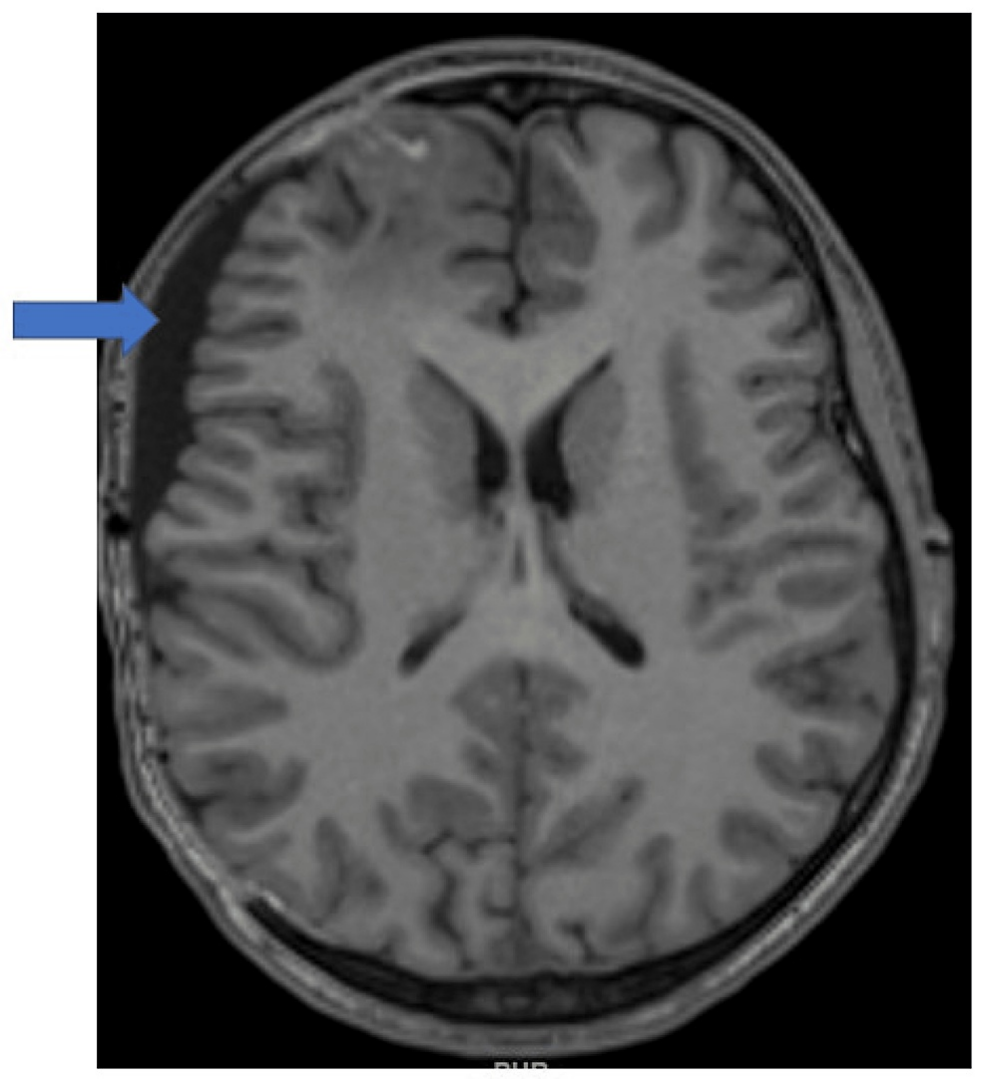
Repeat MRI of the brain at five weeks post-discharge with the craniectomy flap (arrow). MRI: magnetic resonance imaging.

Intraoperative culture from sinus tissue revealed that *Staphylococcus epidermidis* resistant to beta-lactams and *Streptococci viridans* resistant to penicillin. Fungal cultures from sinus and brain abscesses were negative, as were gram stains and cultures from brain abscesses. However, PCR from the brain abscess was positive for *Streptococcus intermedius*. Intravenous vancomycin was continued for six weeks with two weeks of oral metronidazole. The patient was transferred from the intensive care unit on postoperative day four (symptom day 27). He received levetiracetam for seizure prophylaxis for seven days and was discharged on hospital day 14 (symptom day 37). After he was discharged, he was given home intravenous antibiotic therapy for six weeks. The patient was seen in the neurological surgical services clinic at six weeks and with a GCS of 15, no cranial nerve or focal sensorimotor deficit, and a soft and full craniectomy flap (Figure 6). The patient is due to be scheduled for a cranioplasty. We obtained informed consent from the patient's parent and assent from the patient to present and publish the case report. 

## Discussion

While well documented in immunocompromised states after sinusitis, intracranial complications of upper respiratory infections and rhinosinusitis are uncommon in healthy and fully immunized patients [13]. Most healthy patients infected with the SARS-CoV-2 virus recover without antiviral treatments. However, approximately 5% of patients progress to critical forms of pulmonary disease and multi-organ failure and death [14], suggesting that the host’s immune response to the virus could influence SARS-CoV-2 infection outcomes in otherwise healthy patients. We present the first report of an otherwise healthy adolescent who required emergent decompressive craniectomy for a life-threatening brain abscess-related cerebral herniation complicating recent SARS-CoV-2 sinusitis.

Multiple signaling pathways are involved in the interaction between the host immune response and the SARS-CoV-2 virus. The interactions that attenuate the human immune response include nonstructural protein 1 (Nsp1) encoded by SARS-CoV-2 [15-20], which not only suppresses the host immune response but also reduces global gene and human leukocyte antigen-G (HLA-G), which then induces profound immune suppression in SARS-CoV-2-infected patients. This patient exhibited secondary infection, possibly due to a dysregulated host response to severe infection [21].

Tian et al. performed quantitative proteomics analysis of urine samples from COVID-19 patients, healthy controls, and patients with non-COVID-19 pneumonia. They found many immune proteins (145 proteins) are down-regulated in the early stage of SARS-CoV-2 infection. These proteins are involved in the complement and coagulation cascades, natural killer cell-mediated cytotoxicity, platelet activation, tight junction formation, and cell-cell adhesion junctions. They also found some up-regulated proteins associated with the immune response in the late stage of the disease, so they proposed a “two-stage” model of SARS-CoV-2 pathogenesis [22]. The initial stage may involve immune system suppression and damage to tight junctions. The second stage may involve activated immune responses, which cause cytokine release syndrome/cytokine storm and multi-organ damage. Interestingly, the SARS-CoV-2 virus directly infects astrocytes, leading to choroid plexus breakdown, neuronal death, and neurological complications [22,23], and approximately 7.2% of patients have brain fog long after recovery from SARS-CoV-2 infection [24]. Our patient presented with SARS-CoV-2 rhinosinusitis and a bacterial brain abscess, a phenomenon reported to occur in 3% of immunocompetent children admitted for sinusitis treatment [25].

Frontal and anterior ethmoid sinusitis make the frontal skull particularly vulnerable to the spread of infection [26]. Osteomyelitis of the sinus walls or existing bony defects can also lead to infectious intracranial complications. Intracranial brain abscess can develop from the perivascular inflammatory response surrounding necrotic tissue (cerebritis), which expands and forms a capsule [27]. Patients may present with seizures and behavioral changes [26,27], and rarely, as our case demonstrates, life-threatening intracranial hypertension ensues. Frontal brain abscesses are the most common intracranial complication from sinusitis (37%-52%) [26,27] and expand slowly with symptoms occurring over weeks. Abscess sizes greater than 2.5 cm in diameter with extensions into surrounding brain structures can result in brain edema and herniation syndrome necessitating emergency neurosurgical intervention [27]. What is unusual and unclear is that despite craniotomy and abscess drainage, our patient developed a postoperative decline in mental status, bradycardia, and systolic hypertension, consistent with herniation syndrome and needed decompressive craniectomy and whether that is a continued sequela from SARS-CoV-2 or the bacterial infection [28,29]. Prompt diagnosis and treatment of brain abscesses were necessary; equally important was early transfer to a medical center where neurocritical care services are available [30] to detect brain herniation and provide decompressive craniectomy as a life-saving treatment.

The widespread availability of CT has an important role, but MRI is more sensitive than CT for the early diagnosis of brain abscess (positive predictive value 98%, negative predictive value 92%) [13,27,31]. Cultures of the sinuses, blood, and cerebrospinal fluid (CSF) help identify the causative pathogen, which is typically a Streptococcus or Staphylococcus strain [26,27]. Cultures of blood and CSF identify the causative pathogen in about 25% of patients. Neurosurgery, i.e., craniotomy or stereotactic aspiration, is necessary to reduce the abscess size, especially that of more than 2.5 cm in diameter. As a delay in treatment leads to poor outcomes, the recommendation is to begin earlier empirical treatment based on clinical suspicion of a brain abscess [27]. For contiguous spread, empirical treatment includes ceftriaxone, metronidazole, and vancomycin if Staphylococcus is identified and includes antifungals if cultures are positive for fungal species such as Aspergillus [27]. This case illustrates the temporal relationship of brain abscess development despite oral antibiotic therapy and initial CT imaging. 

Given our inability to determine the risk of moderate or severe SARS-CoV-2 infection in healthy children, neurological manifestations in children cannot be ignored. Additionally, the disproportionate disease burden from COVID-19 in marginalized groups, including Black and Hispanic children, requires additional consideration. The pediatric inflammatory, multisystem syndrome (MIS-C) temporally associated with SARS-CoV-2 is a post-infectious hyperinflammatory condition related to SARS-CoV-2 infection (typically weeks after SARS-CoV-2 infection) and associated with a high-frequency of neurological problems like status epilepticus, focal deficits, headache, hallucinations, encephalopathy [8] and one report of fatal cerebral edema [28]. Based on the case series, it is estimated that Black children represent 25-40% of the multisystem inflammatory syndrome (MIS-C) cases in the USA. A multicenter, retrospective and prospective study of 281 pediatric hospitalized patients with acute SARS-CoV-2 infection and MIS-C was divided into three groups based on clinical features (respiratory, MIS-C, and gastrointestinal (GI) or other). The 25% who had MIS-C were more likely to identify as non-Hispanic black than patients with respiratory disease (35% vs. 18%, p=0.02) [32]. Although patients with MIS-C had good outcomes, they were more critically ill, required vasopressors and immunomodulatory therapy, and were more likely than those with respiratory disease to be admitted to the ICU. 

Access to timely prevention and emergent health care is also challenging for patients and communities most affected by the SARS-CoV-2 infection [33]. In this case, the patient received definitive and life-saving care at a safety net hospital where round-the-clock neurosurgical, neurocritical care, and anesthesiology care are available. This case highlights the need for hourly vital signs, including GCS, in patients with brain abscesses that can recognize and act upon cerebral herniation syndrome promptly to have a favorable neurological outcome.

## Conclusions

SARS-CoV-2 infection in children usually presents with mild symptoms. However, some healthy children and adolescents have complications that clinicians are unable to predict. Clinicians should have a high index of suspicion for diagnosing invasive sinusitis and brain abscess in patients with acute neurological manifestations, including life-threatening intracranial hypertension during and after SARS-CoV-2 infection.
